# Genetic and Biochemical Diversity for *N*-acylhomoserine Lactone Biosynthesis in the Plant Pathogen *Pectobacterium carotovorum* subsp. *carotovorum*

**DOI:** 10.1264/jsme2.ME19105

**Published:** 2019-12-27

**Authors:** Tomohiro Morohoshi, Yuto Ogasawara, Xiaonan Xie, Hiroshi Hamamoto, Nobutaka Someya

**Affiliations:** 1 Department of Material and Environmental Chemistry, Graduate School of Engineering, Utsunomiya University 7–1–2 Yoto, Tochigi 321–8585 Japan; 2 Center for Bioscience Research and Education, Utsunomiya University 350 Mine-machi, Utsunomiya, Tochigi 321–8505 Japan; 3 Department of Clinical Plant Science, Faculty of Bioscience and Applied Chemistry, Hosei University 3–7–2 Kajino-cho, Koganei, Tokyo, 184–8584 Japan; 4 Institute of Vegetable and Floriculture Science, National Agriculture and Food Research Organization 3–1–1 Kannondai, Ibaraki 305–8519 Japan

**Keywords:** quorum sensing, acylhomoserine lactone, *Pectobacterium carotovorum* subsp. *carotovorum*, diversity, plant pathogen

## Abstract

The plant pathogen *Pectobacterium carotovorum* subsp. *carotovorum* (*Pcc*) regulates the expression of virulence factors by *N*-acylhomoserine lactone (AHL)-mediated quorum sensing. The LuxI family protein, ExpI, catalyzes AHL biosynthesis in *Pcc*. The structure of the predominant AHL produced by ExpI differs among *Pcc* strains, which may be divided into two quorum-sensing classes (QS classes) based on the AHL produced. In the present study, AHL produced by 282 *Pcc* strains were extracted and identified by LC-MS/MS. Seventy *Pcc* strains produced *N*-(3-oxooctanoyl)-l-homoserine lactone (3-oxo-C8-HSL) as the predominant AHL and were categorized into QS class I. Two hundred *Pcc* strains produced *N*-(3-oxohexanoyl)-l-homoserine lactone (3-oxo-C6-HSL) as the predominant AHL, and were categorized into QS class II-1. Twelve *Pcc* strains produced only small amounts of 3-oxo-C6-HSL, and were categorized into QS class II-2. The phylogenetic analysis revealed that the amino acid sequences of ExpI may be divided into two major clades (I and II). The *Pcc* strains categorized into ExpI clades I and II entirely matched QS classes I and II, respectively. A multiple alignment analysis demonstrated that only 6 amino acid substitutions were observed among ExpI from QS classes II-1 and II-2. Furthermore, many amino acid substitutions between QS classes I and II were concentrated at the C-terminal region. These amino acid substitutions are assumed to cause significant reductions in 3-oxo-C6-HSL in QS class II-2 or affect the substrate specificity of ExpI between QS classes I and II.

Quorum sensing is one of the bacterial cell-to-cell communication processes that regulate gene expression in response to increases in cell density (13). In quorum sensing, bacteria release chemical signaling molecules called autoinducers (AI) and recognize their own cell density as the concentration of AI (13). In many Gram-negative bacteria, *N*-acyl-l-homoserine lactone (AHL) is mainly used as AI (19). LuxI family proteins catalyze the biosynthesis of AHL from *S*-adenosyl-l-methionine and acyl-acyl carrier proteins or CoA–aryl/acyl moieties (6). AHL binds to LuxR family proteins and the AHL-LuxR complex controls the transcription of many genes responsible for bioluminescence, the production of pigment, or the production of antibiotics (19). AHL-mediated quorum sensing is highly conserved in Gram-negative plant pathogenic bacteria (22). A wide range of phenotypes, which include motility, biofilm formation, colonization, and the production of virulence factors, such as extracellular polysaccharides (EPS), surfactants, and extracellular enzymes, are affected by AHL-mediated quorum sensing in plant pathogenic bacteria (22).

Bacterial soft rot is the most serious disease of many economically important plants worldwide and is caused by multiple genera of bacteria, particularly those belonging to the genera *Pectobacterium* and *Dickeya* (1). *Pectobacterium carotovorum* subsp. *carotovorum* (*Pcc*; formerly *Erwinia carotovora* subsp. *carotovora*) is a well-known plant pathogen that causes severe soft rot disease. *Pcc* produces various types of plant cell wall-degrading enzymes (PCWDEs) as major virulence factors, *i.e*. pectate lyases, polygalacturonases, cellulases, and proteases (10). In the quorum-sensing system of *Pcc*, the LuxI homolog (ExpI) catalyzes AHL biosynthesis, while the LuxR homolog (ExpR) binds AHL and regulates the transcription of various virulence genes including PCWDE (20). Since the inactivation of *expI* results in the disappearance of AHL production, decreases in the production of PCWDE, and reduced virulence, AHL is expected to become an important target in the development of a control method for *Pcc* infection (7).

The structure of the predominant AHL produced by ExpI differs among each *Pcc* strain. Põllumaa *et al*. demonstrated that *Pcc* strains may be divided into two quorum-sensing classes (QS classes) based on the AHL produced (20). QS class I and II strains synthesize *N*-(3-oxooctanoyl)-l-homoserine lactone (3-oxo-C8-HSL) and *N*-(3-oxohexanoyl)-l-homoserine lactone (3-oxo-C6-HSL) as the predominant AHL, respectively. The alignment of the predicted amino acid sequences revealed that the sequences of ExpI share more than 90% identity among QS class I strains, but showed lower identity (approximately 70%) between QS class I and II strains (20). The ExpR of class I bind to 3-oxo-C8-HSL and stimulate the transcription of quorum-sensing regulated genes, but not 3-oxo-C6-HSL. In contrast, the ExpR of class II bind to 3-oxo-C6-HSL, but not to 3-oxo-C8-HSL (2). However, since these classifications have been limited to selected *Pcc* strains, the diversity and distribution of QS classes and amino acid sequences of ExpI in *Pcc* culture collections isolated from a number of diseased crops have not yet been elucidated. A number of *Pcc* strains have been isolated from diseased crops and deposited in the NARO Genebank (Tsukuba, Japan) or NITE Biological Resource Center (NBRC; Chiba, Japan). In the present study, we investigated the diversity and distribution of the QS classes and ExpI sequences in the *Pcc* strains in the above culture collections.

## Materials and Methods

### Bacterial strains, compounds, and growth conditions

A total of 282 strains of soft rot-causing bacteria, which were deposited as *Pcc* or *E. carotovora* subsp. *carotovora*, were obtained from NARO Genebank or NBRC and listed in Table S1. *Pcc* strains were grown in trypticase soy broth (TSB; Becton, Dickinson and Sparks, MD, USA) at 30°C. *Escherichia coli* DH5α was grown at 37°C in Luria-Bertani (LB) medium at 37°C. Two AHL reporters, *Chromobacterium violaceum* CV026 (12) and VIR07 (14), were grown in LB or TSB medium at 30°C. Solid bacterial media were made by adding agar at a final concentration of 1.5%. Ampicillin was added as required at a final concentration of 100 μg mL^−1^.

### Extraction, identification, and quantification of AHL molecules

*Pcc* strains were inoculated onto TSB agar medium prepared in 24-well plates. Two AHL reporter strains, *C. violaceum* CV026 and VIR07, were also inoculated onto the lower right or left positions of the well. After an incubation at 30°C for 2 d, AHL-producing activity was detected as the induced production of purple pigments by the AHL reporter strains. Regarding the extraction of AHL from the culture supernatant, *Pcc* strains were inoculated into 4 mL of TSB liquid medium. After an incubation for 18 h, bacterial cultures were inoculated into 4 mL of fresh TSB medium (1% inoculum) and incubated for 20 h. Seven hundred microliters of the culture supernatant was mixed with an equal volume of ethyl acetate and vortexed for 5 min. After centrifugation, 600 μL of the ethyl acetate layer was transferred to the new microtube, evaporated to dryness, and dissolved in 100 μL dimethylsulfoxide. The identification and quantification of AHL molecules by mass spectrometry were performed by Liquid Chromatography-Tandem Mass Spectrometry (LC-MS/MS) as described previously (15–17). The AHL standards, *N*-hexanoyl-l-homoserine lactone (C6-HSL), *N*-octanoyl-l-homoserine lactone (C8-HSL), 3-oxo-C6-HSL, 3-oxo-C8-HSL, and *N*-(3-oxodecanoyl)-l-homoserine lactone (3-oxo-C10-HSL) were synthesized using a previously described method (3).

### Identification and sequencing of the *expI* gene in *Pcc* strains

The genomic DNA of *Pseudomonas* strains was extracted using the DNeasy Blood & Tissue Kit (Qiagen, Tokyo, Japan). The specific primers for PCR amplification of the *expI* homolog, expI-F1 (5′-CTGCGTCGTGGTAATGATTACTCCATCATG-3′), expI-F2 (5′-TGAACTCTTGCTGACAATGCAGGTTGCATCTGTAC-3′), expI-R1 (5′-ACAGCACGATTGACGCCAGCTATGACAGAG-3′), and expI-R2 (5′-CGCTACAGCCGCGCCTACATTAAAGACAG-3′) were designed based on the complete genome sequence of *Pcc* PCC21 (18). PCR was performed with KOD FX Neo DNA polymerase (Toyobo, Osaka, Japan) using the following cycling parameters: 98°C for 10 s, 55°C for 30 s, and 68°C for 2 min for 30 cycles. PCR products were separated by electrophoresis, purified using NucleoSpin Gel and PCR Clean-up (Takara Bio, Shiga, Japan), and sequenced using the BigDye Terminator v3.1 Cycle Sequencing Kit and 3500 Series Genetic Analyzer (Applied Biosystems, Tokyo, Japan). The phylogenetic tree of the amino acid sequences of ExpI was constructed using the neighbor-joining method with 1,000 bootstrap replicates by MEGA 7 software (9). ExpI sequences were aligned using the ClustalW program (21) and shaded using GeneDoc software.

### Cloning and characterization of the *expI* gene from *Pcc* strains

The specific primers, expI-F2 and expI-R3 (5′-CAGCACGGTA AAATTTCATATCGGCAACGTTGTG-3′) were used to amplify the *expI* gene. PCR was performed with Blend Taq Plus DNA polymerase (Toyobo) using the following cycling parameters: 94°C for 30 s, 60°C for 30 s, and 72°C for 1.5 min for 30 cycles. PCR products were separated by electrophoresis and then purified using NucleoSpin Gel and PCR Clean-up. The purified PCR products were cloned into the pGEM-T easy cloning vector (Promega, Tokyo, Japan). The constructed plasmid was sequenced to establish whether the *expI* gene was located downstream of the *lac* promoter on the vector. *E. coli* DH5α harboring the *expI*-expressing plasmid was inoculated into 4 mL of LB medium and incubated for 18 h. The *E. coli* culture was inoculated into 4 mL of fresh TB medium (1% inoculum) and incubated for 20 h. AHL molecules in the culture supernatant were identified and quantified by LC-MS/MS using the above-described method.

### Infectivity assay on Chinese cabbage

The *Pcc* strains grown on nutrient agar were suspended in sterilized water (10^7^–10^8^ cells mL^−1^) and used as an inoculum. The suspensions were mixed with or without 3-oxo-C6-HSL solutions to obtain a final concentration of 25 μM. The surfaces of Chinese cabbage leaves were gently washed with disinfecting foaming soap and rinsed thoroughly with tap water. The surfaces were then picked by an autoclaved toothpick and used for the infectivity assay. Bacterial suspensions with or without 3-oxo-C6-HSL were placed onto the picked holes on the leaves. Inoculated samples were incubated at 25°C and images of the leaves were taken every 3 min using a web camera to monitor the onset and development of symptoms.

### Nucleotide sequence accession number

The nucleotide sequences of the *expI* genes of 279 *Pcc* strains obtained from NARO Genebank were deposited under DDBJ/ENA/GenBank accession numbers LC386966 to LC387244. The nucleotide sequences of *expI* genes from NBRC 3380, 3830, and 12380 were deposited under accession numbers LC387836, LC386957, and LC386958, respectively.

## Results and Discussion

### Characterization of AHL produced by *Pcc* strains

**Fig. 1:**
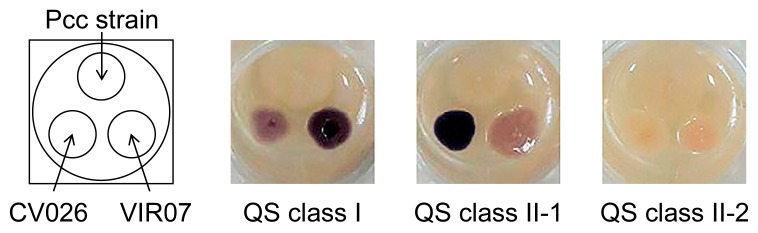
AHL-producing activity of *Pcc* strains. Bacterial strains were inoculated onto TSB agar medium prepared in 24-well plates. Two AHL reporter strains, *C. violaceum* CV026 and VIR07, were also inoculated onto the lower left and right positions of the well, respectively. After an incubation at 30°C for 40 h, AHL-producing activity was detected as the induced production of purple pigments by the AHL reporter strains and classified into QS classes I, II-1, and II-2.

In the present study, we used 282 *Pcc* strains obtained from NARO Genebank and NBRC. The AHL-producing activities of *Pcc* strains were checked by a cross-feeding assay using the AHL biosensors *C. violaceum* CV026 and VIR07. The AHL production patterns of *Pcc* strains were classified into three types based on the induction of violacein production in *C. violaceum* CV026 and VIR07 (Fig. 1). The AHL production patterns of all 282 *Pcc* strains were listed in Table S1. The 70 *Pcc* strains of AHL production type I strongly induced violacein production in VIR07, but weakly in CV026. The 200 *Pcc* strains of AHL production type II strongly induced violacein production in CV026, but weakly in VIR07. The 12 *Pcc* strains of AHL production type IIII did not induce violacein production in CV026 or VIR07. AHL was then extracted from the culture supernatant of 282 strains. HPLC fractionation followed by a MS analysis of AHL extracts was performed to identify the structure and production ratio of AHL from the results of the peak area ratio (Table S2). In the AHL extracts of the 70 *Pcc* strains of AHL production type I, 3-oxo-C8-HSL was identified as the predominant AHL and 3-oxo-C6-HSL, C6-HSL, C8-HSL, and 3-oxo-C10-HSL were detected as minor AHL. These AHL production profiles, which contained 3-oxo-C8-HSL as a major AHL, corresponded to those of the previously reported class I quorum-sensing system (20). Thus, we classified AHL production type I strains into QS class I. In the AHL extracts of the 200 *Pcc* strains of AHL production type II, 3-oxo-C6-HSL was detected as the predominant AHL and C6-HSL, 3-oxo-C8-HSL, and C8-HSL were minor AHL. 3-Oxo-C6-HSL and C6-HSL accounted for approximately 97%. A small amount of 3-oxo-C6-HSL was present in the AHL extracts of the 12 *Pcc* strains of AHL production type III. The small amount of 3-oxo-C6-HSL produced by the *Pcc* strains of AHL production type III did not appear to reach the threshold for the induction of violacein production in CV026 or VIR07. These AHL production profiles, which contain 3-oxo-C6-HSL as a major AHL, corresponded to those of a previously reported class II quorum-sensing system (20). Thus, we classified AHL production types II and III into QS classes II-1 and II-2, respectively. In the LC-MS/MS analysis, AHL molecules were detected by screening samples for the precursor ions that gave rise to a fragment ion at *m*/*z* 102 (16). However, AHLs, except for those described above, were not detected from the samples extracted from the *Pcc* strains used in the present study (data not shown).

### Phylogenetic diversity of ExpI in *Pcc* strains

**Fig. 2:**
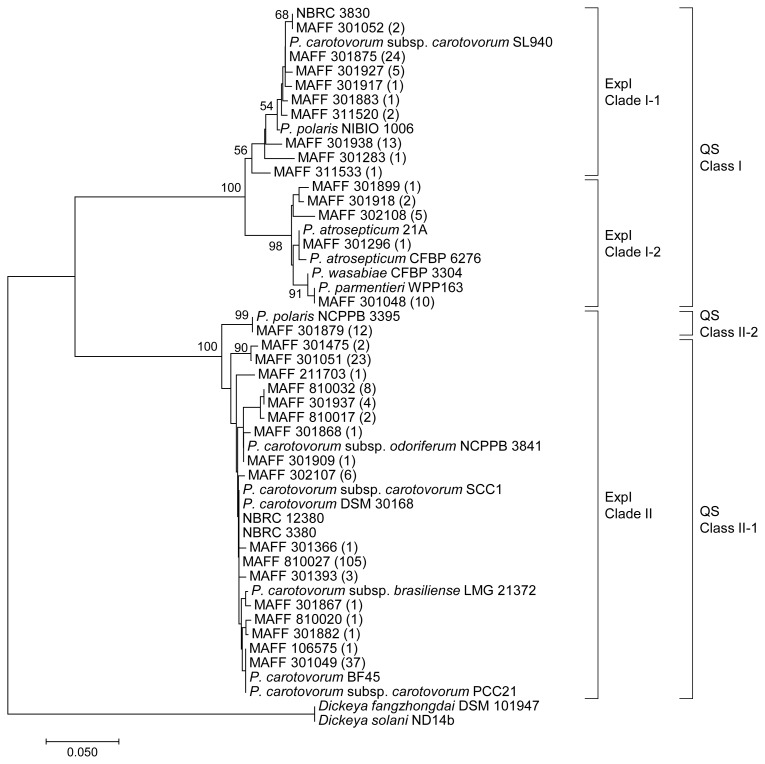
Phylogenetic tree based on amino acid sequences of ExpI from 282 *Pcc* strains. The phylogenetic tree was constructed using the neighbor-joining method with the ClustalW program of MEGA7. The percentage of replicate trees in which the associated taxa clustered together in the bootstrap test (1,000 replicates) is shown next to the branches. The scale bar represents 0.05 substitutions per amino acid position. LuxI from *Dickeya fangzhongdai* DSM 101947 and *Dickeya solani* ND14b were used as outgroups. The phylogenetic classes of LuxI homologs (QS class and ExpI clade) were described on the right side of the tree. The number of identical sequences was represented in the parentheses.

In *Pectobacterium*, AHL is synthesized by a single LuxI homologue called ExpI (20). To confirm whether the *expI* gene homolog was present in the genomes of 282 *Pcc* strains, specific primers were designed based on the complete genome sequence of *Pcc* PCC21 (18). DNA fragments were successfully amplified from the genomes of all *Pcc* strains by using the above specific primers. The specific primer, expI-F2, was used to sequence the complete *expI* sequence. The deduced amino acid sequences of ExpI were classified with multiple sequence alignments. Based on the results of the phylogenetic analysis, ExpI from *Pcc* strains were divided into two major sequence clades at an identity level of 75% (Fig. 2). ExpI clade I contained 70 strains and these strains completely corresponded to the classification of QS class I. Furthermore, it was possible to divide ExpI clade I into two subclades (ExpI clade I-1 and I-2) at an identity level of 95%. ExpI clade II contained 212 strains that belonged to QS class II-1 or II-2. The amino acid sequences of ExpI from the 12 strains belonging to QS class II-2 completely matched each other and showed higher identity (over 93%) with those belonging to QS class II-1.

A number of amino acid sequences of ExpI homologs have been deposited in the UniProt database. The ExpI homologs identified in the present study showed high similarity with those from various *Pectobacterium* species, *i.e. P. polaris*, *P*. *atrosepticum*, *P. wasabiae*, and *P. parmentieri*. ExpI clade I showed higher identity to the ExpI homologs from *Pectobacterium* species other than *P. carotovorum*. In contrast, ExpI clade II showed higher identity to the ExpI homologs from various subspecies of *P. carotovorum*. ExpI from QS class II-2 showed 100% identity with that from *P*. *polalis* NCPPB 3396, but lower identity (approximately 73%) with that from *P. polalis* NIBIO1006. These results suggest the presence of a significant relationship between the amino acid sequences of ExpI and classification of the *Pectobacterium* species.

### Quantification of AHL produced by *Pcc* strains

To compare AHL production by *Pcc* strains classified to different QS classes and ExpI clades, we selected four representative strains, MAFF 301875 (QS class I and ExpI clade I-1), MAFF 301048 (QS class I and ExpI clade I-2), MAFF 301393 (QS class II-1 and ExpI clade II), and MAFF 301879 (QS class II-2 and ExpI clade II). Extracts of the culture supernatants of these strains were quantified by LC-MS/MS (Table 1). Two QS class I strains, MAFF 301875 and MAFF 301048, mainly produced 3-oxo-C8-HSL at a rate of more than 80% and the sum of 3-oxo-C8-HSL and C8-HSL accounted for approximately 90%. These strains also produced C6-HSL, 3-oxo-C6-HSL, and 3-oxo-C10-HSL as minor products. Although AHL produced by ExpI clade I-2 contained a higher concentration of C6-HSL than that produced by ExpI clade I-1, the whole composition of AHL did not significantly differ between ExpI clades I-1 and I-2 (Table 1). The QS class II-1 strain, MAFF 301393, mainly produced 3-oxo-C6-HSL at a rate of more than 90% and small amounts of C6-HSL and 3-oxo-C8-HSL. These AHL production ratios of *Pcc* strains corresponded to previously reported *Pcc* and its related genus *Pectobacterium* (4, 5). In contrast, the amount of 3-oxo-C6-HSL produced by the QS class II-2 strain was approximately 1/30 that by QS class II-1. The concentration of 3-oxo-C6-HSL produced by QS class II-2 did not appear to reach the threshold to induce violacein production by AHL reporter strains.

### Amino acid substitutions in ExpI affect the structure of AHL

**Fig. 3:**
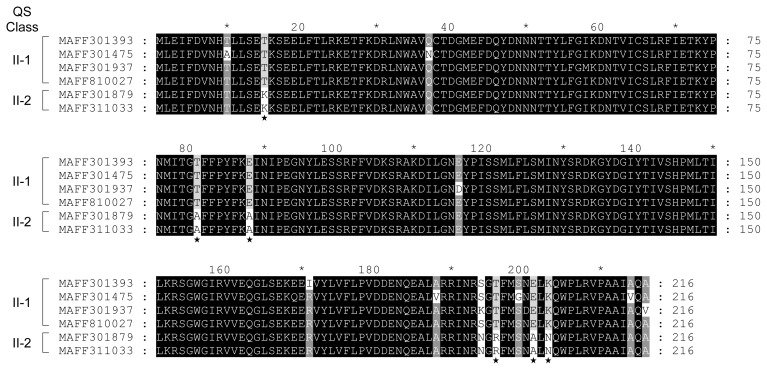
Comparison of amino acid sequences of ExpI from QS class II-1 (MAFF 301785, 301475, 301937, and 810027) and II-2 (MAFF 301879 and 311033). Sequences were aligned using ClustalW and shaded using GeneDoc software. Differences in amino acid sequences between QS classes II-1 and II-2 were indicated by asterisks.

**Fig. 4:**
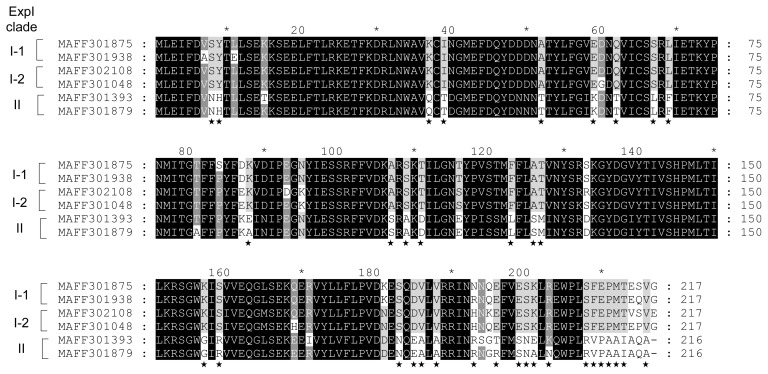
Comparison of amino acid sequences of ExpI clades I-1 (MAFF 301875 and 301938), I-2 (MAFF 302108 and 301048), and II (MAFF 301393 and 301879). Sequences were aligned using ClustalW and shaded using GeneDoc software. Differences in amino acid sequences between ExpI clades I and II were indicated by asterisks.

Amino acid substitutions in ExpI from QS classes II-1 and II-2 were predicted by the ClustalW program. Although QS class II strains produce 3-oxo-C6-HSL as the predominant AHL, QS class II-2 strains produce lower amounts of 3-oxo-C6-HSL than QS class II-1. In the results of multiple alignments between ExpI from four QS class II-1 strains (MAFF 301393, 301475, 301937, and 810027) and two QS class II-2 strains (MAFF 301879 and 311033), only 6 amino acid substitutions were observed among ExpI from QS classes II-1 and II-2 (Fig. 3). Slight amino acid substitutions may significantly reduce the activity of 3-oxo-C6-HSL biosynthesis in ExpI from QS class II-2. The amino acid substitutions in ExpI from QS class I-1 (MAFF 301875 and 301938), I-2 (MAFF 302108 and 301048), and II (MAFF 301393 and 301879) were shown in Fig. 4. The multiple alignment analysis revealed that although some amino acid substitutions were scattered all over the sequences between QS classes I and II, many amino acid substitutions were concentrated at the C-terminal region. These results suggest that these amino acid substitutions affect the substrate specificity of ExpI.

### Structure of AHL produced by heterologously expressed ExpI in *E. coli*

To elucidate the AHL-producing activity of ExpI from *Pcc* strains in more detail, the *expI* genes from the four selected representative *Pcc* strains were amplified by PCR and cloned downstream of the *lac* promoter in the pGEM-T easy vector. Extracts of the culture supernatants of *E. coli* harboring the *expI* gene from *Pcc* strains were quantified by LC-MS/MS (Table 2). ExpI from two QS class I strains, MAFF 301875 and MAFF 301048, produced 3-oxo-C8-HSL as the predominant AHL. In contrast, ExpI from two QS class II strains, MAFF 301393 and MAFF 301879, produced 3-oxo-C6-HSL as the predominant AHL. These features corresponded to the results of the quantification of AHL in the culture extracts of *Pcc* parent strains (Table 1). Small concentrations of C6-HSL and C8-HSL were detected in the samples of *E. coli* harboring the *expI* gene from MAFF 301879 (QS class II-2) and MAFF 301393 (QS class II-1), respectively. Structural differences between the AHL produced in *Pcc* strains and *E. coli* were attributed to the intracellular composition of fatty acids. In addition to the results of the quantification of AHL extracted from the culture of the *Pcc* parent strain, the concentration of 3-oxo-C6-HSL in the sample from *E. coli* expressing ExpI from QS class II-2 was still at a lower level than that from *E*. *coli* expressing ExpI from QS class II-1. These results demonstrated that the low-level production of 3-oxo-C6-HSL in QS class II-2 was not due to the transcriptional level of *expI*, but to differences in the amino acid sequence of ExpI between QS classes II-1 and II-2.

### Comparison of the pathogenicity of *Pcc* strains with different AHL-producing activities

In order to understand the relationship between AHL-producing activity and pathogenicity, we conducted pathogenicity tests on Chinese cabbage leaves. Time-course of progression symptom was compared between MAFF 301875 (normal AHL-producing strain) and MAFF 301879 (AHL-decreasing strain). Although the onset of symptom development in MAFF 301879 was later than that in MAFF 301875, no significant differences were observed in increasing rate of duration of the symptoms (Fig. S1). These results suggest that due to low AHL production, MAFF 301879 more slowly activates the quorum-sensing system. The complementary effect of exogenous AHL was assessed using the MAFF 301879 strain. The addition of 3-oxo-C6-HSL appeared to partially complement delayed symptom development; however, the initiation point of symptom development did not appear to be stable in the present study (data not shown). The initial activation of quorum sensing at the stage of low cell density may destabilize the regulated expression of virulence factors.

## Conclusion

In the present study, we demonstrated that the *Pcc* strains deposited in culture collections may be clearly categorized into two QS classes and two sequence clades of ExpI. Soft rot bacteria, which were formerly classified as *E. carotovora*, have been continuously reclassified by various methods, such as 16S rRNA sequencing, O serogroups, and DNA–DNA hybridization (8). A recent comparative genome analysis revealed that 84 *Pectobacterium* strains were classified into *P. carotovorum*, *P. parmentieri*, *P. atrosepticum*, *P. polaris*, and Candidatus *Pectobacterium maceratum* (11). In that study, the maximum likelihood tree based on SNP data indicated that the whole genome sequences of *P. polaris* NIBIO 1006 and NCPPB 3395 showed high similarities and were classified into the same group (11). However, although the amino acid sequence of ExpI from *P. polaris* NCPPB 3395 showed 100% identity to that of QS class II-2, ExpI from *P. polaris* NIBIO 1006 was categorized into another QS class I (Fig. 2). These results suggest that the diversity of quorum sensing-related genes is applicable to the definition of new intraspecific names for serovars, biovars, and pathovars.

## SUPPLEMENTARY MATERIAL



## Figures and Tables

**Table 1:** Quantification of AHL produced by*Pcc* strains

Strains	MAFF 301875	MAFF 301048	MAFF 301393	MAFF 301879
QS class	I	I	II-1	II-2

ExpI clade	I-1	I-2	II	II

C6-HSL	2.6±0.16	129±8	31±2.5	—
C8-HSL	236±1.6	151±2.9	—	—
3-oxo-C6-HSL	37±2.0	25±0.33	464±36	15±3.9
3-oxo-C8-HSL	1,846±27	1,444±37	3.6±0.46	—
3-oxo-C10-HSL	32±1.0	26±1.2	—	—

AHL concentrations are expressed in nM.

—, Below the detection limit.

**Table 2:** Quantification of AHL produced by*E. coli* harboring the *expI* gene from *Pcc* strains

*expI* source	MAFF 301875	MAFF 301048	MAFF 301393	MAFF 301879
QS class	I	I	II-1	II-2

ExpI clade	I-1	I-2	II	II

C6-HSL	4.6±0.20	33±1.9	400±21	18±0.22
C8-HSL	241±15	1,380±40	4.4±0.30	[Table-fn tfn4-34_429]—
3-oxo-C6-HSL	25±2.1	158±12	1,379±35	96±0.62
3-oxo-C8-HSL	2,119±87	5,103±155	19±1.7	[Table-fn tfn4-34_429]—
3-oxo-C10-HSL	37±2.2	296±26	[Table-fn tfn4-34_429]—	[Table-fn tfn4-34_429]—

AHL concentrations are expressed in nM.

—, Below the detection limit.
