# Supplementary material for: Genetic and Biochemical Diversity for N-acylhomoserine Lactone Biosynthesis in the Plant Pathogen Pectobacterium carotovorum subsp. carotovorum
Source: Microbes Environ. 2019 Dec 27;34(4):429–35. doi: 10.1264/jsme2.ME19105 (PMC6934387; doi:10.1264/jsme2.ME19105)
Supplement: Supplementary file 1 [file 34_429_s1.pdf]

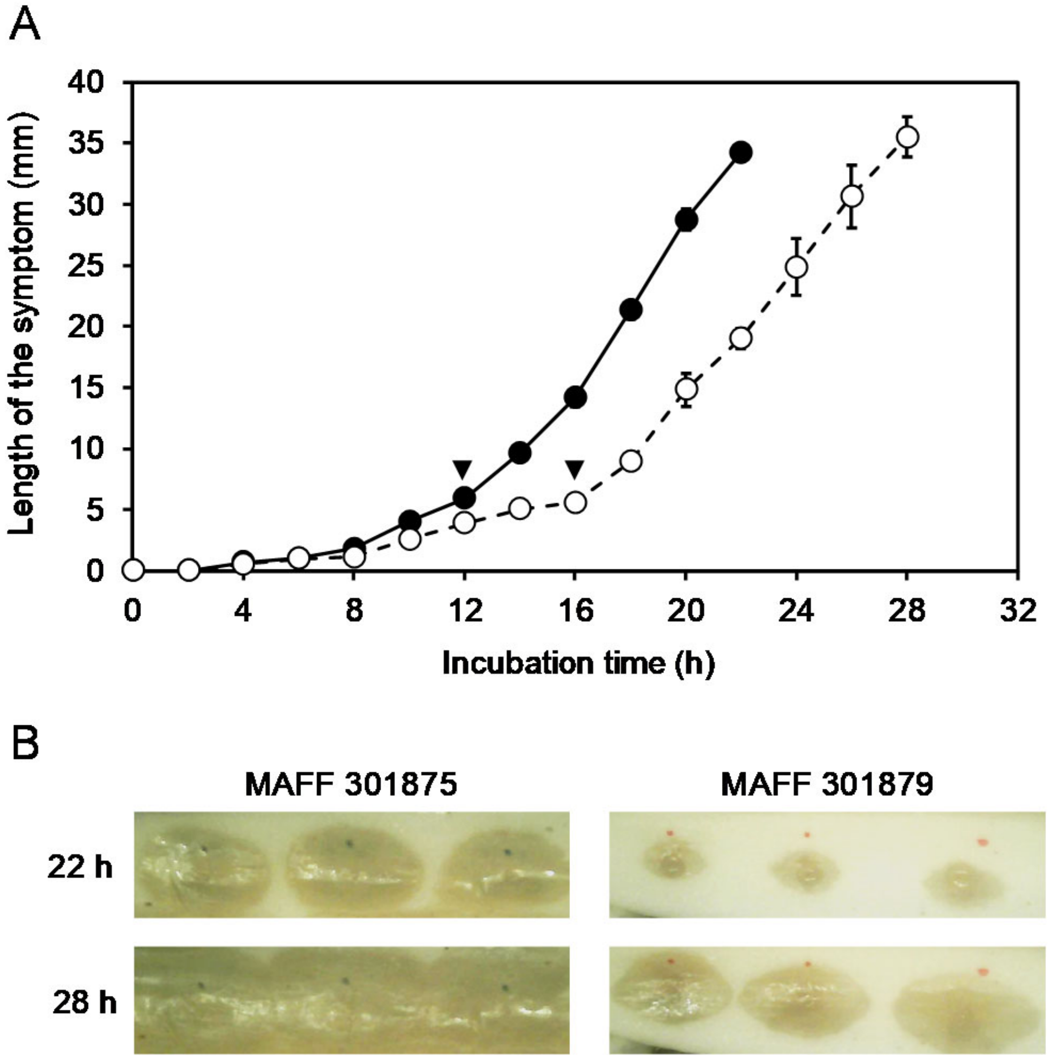


**Fig. S1.** Typical results of the pathogenicity tests on the Chinese cabbage leaves of Pcc strains. (A) Development of the soft rot symptom calculated using the time-laps images, MAFF 301875 (solid line) and MAFF 301879 (dotted line). Note that the onset of the symptom development (arrowhead) of was MAFF 301879 delayed. (B) Images of the developed symptom of both strains.

Table S1. Characterization and classification of AHL biosynthesis of *Pcc* strains

| Strain | Cross streak assay [a] | | | QS class [b] | ExpI clade [c] | Accession no. |
|---|---|---|---|---|---|---|
| | CV026 | VIR07 | AHL type | | | |
| NBRC 3380 | ++ | + | II | II-1 | II | LC387836 |
| NBRC 3830 | ++ | + | II | I | I | LC386957 |
| NBRC 12380 | + | ++ | I | II-1 | II | LC386958 |
| MAFF 106567 | + | ++ | I | I | I | LC386966 |
| MAFF 106568 | + | ++ | I | I | I | LC386967 |
| MAFF 106569 | + | ++ | I | I | I | LC386968 |
| MAFF 106570 | + | ++ | I | I | I | LC386969 |
| MAFF 106571 | + | ++ | I | I | I | LC386970 |
| MAFF 106572 | + | ++ | I | I | I | LC386971 |
| MAFF 106573 | + | ++ | I | I | I | LC386972 |
| MAFF 106574 | + | ++ | I | I | I | LC386973 |
| MAFF 106575 | ++ | + | II | II-1 | II | LC386974 |
| MAFF 106576 | + | ++ | I | I | I | LC386975 |
| MAFF 106664 | ++ | + | II | II-1 | II | LC386976 |
| MAFF 106665 | ++ | + | II | II-1 | II | LC386977 |
| MAFF 211376 | – | – | III | II-2 | II | LC386978 |
| MAFF 211377 | – | – | III | II-2 | II | LC386979 |
| MAFF 211378 | – | – | III | II-2 | II | LC386980 |
| MAFF 211379 | + | ++ | I | I | I | LC386981 |
| MAFF 211380 | + | ++ | I | I | I | LC386982 |
| MAFF 211381 | ++ | + | II | II-1 | II | LC386983 |
| MAFF 211382 | + | ++ | I | I | I | LC386984 |
| MAFF 211383 | + | ++ | I | I | I | LC386985 |
| MAFF 211384 | + | ++ | I | I | I | LC386986 |
| MAFF 211385 | + | ++ | I | I | I | LC386987 |
| MAFF 211386 | – | – | III | II-2 | II | LC386988 |
| MAFF 211703 | ++ | + | II | II-1 | II | LC386989 |
| MAFF 212078 | ++ | + | II | II-1 | II | LC386990 |
| MAFF 212079 | ++ | + | II | II-1 | II | LC386991 |
| MAFF 212080 | ++ | + | II | II-1 | II | LC386992 |
| MAFF 212081 | ++ | + | II | II-1 | II | LC386993 |
| MAFF 212082 | ++ | + | II | II-1 | II | LC386994 |
| MAFF 212083 | ++ | + | II | II-1 | II | LC386995 |
| MAFF 301048 | + | ++ | I | I | I | LC386996 |
| MAFF 301049 | ++ | + | II | II-1 | II | LC386997 |
| MAFF 301050 | ++ | + | II | II-1 | II | LC386998 |
| MAFF 301051 | ++ | + | II | II-1 | II | LC386999 |
| MAFF 301052 | + | ++ | I | I | I | LC387000 |
| MAFF 301053 | ++ | + | II | II-1 | II | LC387001 |
| MAFF 301054 | + | ++ | I | I | I | LC387002 |
| MAFF 301056 | ++ | + | II | II-1 | II | LC387003 |
| MAFF 301281 | ++ | + | II | II-1 | II | LC387004 |
| MAFF 301282 | ++ | + | II | II-1 | II | LC387005 |
| MAFF 301283 | + | ++ | I | I | I | LC387006 |
| MAFF 301296 | + | ++ | I | I | I | LC387007 |
| MAFF 301297 | ++ | + | II | II-1 | II | LC387008 |
| MAFF 301298 | ++ | + | II | II-1 | II | LC387009 |
| MAFF 301299 | ++ | + | II | II-1 | II | LC387010 |
| MAFF 301300 | ++ | + | II | II-1 | II | LC387011 |
| MAFF 301301 | ++ | + | II | II-1 | II | LC387012 |

| | | | | | | |
|---|---|---|---|---|---|---|
| MAFF 301362 | ++ | + | II | II-1 | II | LC387013 |
| MAFF 301363 | ++ | + | II | II-1 | II | LC387014 |
| MAFF 301364 | ++ | + | II | II-1 | II | LC387015 |
| MAFF 301365 | ++ | + | II | II-1 | II | LC387016 |
| MAFF 301366 | ++ | + | II | II-1 | II | LC387017 |
| MAFF 301391 | ++ | + | II | II-1 | II | LC387018 |
| MAFF 301392 | ++ | + | II | II-1 | II | LC387019 |
| MAFF 301393 | ++ | + | II | II-1 | II | LC387020 |
| MAFF 301394 | + | ++ | I | I | I | LC387021 |
| MAFF 301395 | ++ | + | II | II-1 | II | LC387022 |
| MAFF 301396 | + | ++ | I | I | I | LC387023 |
| MAFF 301397 | ++ | + | II | II-1 | II | LC387024 |
| MAFF 301398 | ++ | + | II | II-1 | II | LC387025 |
| MAFF 301399 | + | ++ | I | I | I | LC387026 |
| MAFF 301400 | + | ++ | I | I | I | LC387027 |
| MAFF 301401 | ++ | + | II | II-1 | II | LC387028 |
| MAFF 301402 | ++ | + | II | II-1 | II | LC387029 |
| MAFF 301403 | ++ | + | II | II-1 | II | LC387030 |
| MAFF 301404 | ++ | + | II | II-1 | II | LC387031 |
| MAFF 301405 | ++ | + | II | II-1 | II | LC387032 |
| MAFF 301475 | ++ | + | II | II-1 | II | LC387033 |
| MAFF 301476 | ++ | + | II | II-1 | II | LC387034 |
| MAFF 301477 | ++ | + | II | II-1 | II | LC387035 |
| MAFF 301478 | ++ | + | II | II-1 | II | LC387036 |
| MAFF 301479 | ++ | + | II | II-1 | II | LC387037 |
| MAFF 301480 | ++ | + | II | II-1 | II | LC387038 |
| MAFF 301481 | ++ | + | II | II-1 | II | LC387039 |
| MAFF 301482 | ++ | + | II | II-1 | II | LC387040 |
| MAFF 301483 | ++ | + | II | II-1 | II | LC387041 |
| MAFF 301484 | ++ | + | II | II-1 | II | LC387042 |
| MAFF 301618 | + | ++ | I | I | I | LC387043 |
| MAFF 301619 | + | ++ | I | I | I | LC387044 |
| MAFF 301620 | + | ++ | I | I | I | LC387045 |
| MAFF 301645 | ++ | + | II | II-1 | II | LC387046 |
| MAFF 301646 | ++ | + | II | II-1 | II | LC387047 |
| MAFF 301647 | ++ | + | II | II-1 | II | LC387048 |
| MAFF 301648 | ++ | + | II | II-1 | II | LC387049 |
| MAFF 301649 | ++ | + | II | II-1 | II | LC387050 |
| MAFF 301650 | ++ | + | II | II-1 | II | LC387051 |
| MAFF 301651 | ++ | + | II | II-1 | II | LC387052 |
| MAFF 301652 | ++ | + | II | II-1 | II | LC387053 |
| MAFF 301653 | ++ | + | II | II-1 | II | LC387054 |
| MAFF 301654 | ++ | + | II | II-1 | II | LC387055 |
| MAFF 301655 | ++ | + | II | II-1 | II | LC387056 |
| MAFF 301656 | ++ | + | II | II-1 | II | LC387057 |
| MAFF 301865 | ++ | + | II | II-1 | II | LC387058 |
| MAFF 301867 | ++ | + | II | II-1 | II | LC387059 |
| MAFF 301868 | ++ | + | II | II-1 | II | LC387060 |
| MAFF 301869 | ++ | + | II | II-1 | II | LC387061 |
| MAFF 301871 | ++ | + | II | II-1 | II | LC387062 |
| MAFF 301872 | ++ | + | II | II-1 | II | LC387063 |
| MAFF 301873 | ++ | + | II | II-1 | II | LC387064 |
| MAFF 301874 | ++ | + | II | II-1 | II | LC387065 |
| MAFF 301875 | + | ++ | I-1 | I | I-1 | LC387066 |

| | | | | | | |
|---|---|---|---|---|---|---|
| MAFF 301876 | ++ | + | II | II-1 | II | LC387067 |
| MAFF 301877 | ++ | + | II | II-1 | II | LC387068 |
| MAFF 301878 | ++ | + | II | II-1 | II | LC387069 |
| MAFF 301879 | – | – | III | II-2 | II | LC387070 |
| MAFF 301880 | ++ | + | II | II-1 | II | LC387071 |
| MAFF 301881 | – | – | III | II-2 | II | LC387072 |
| MAFF 301882 | ++ | + | II | II-1 | II | LC387073 |
| MAFF 301883 | + | ++ | I | I | I | LC387074 |
| MAFF 301884 | ++ | + | II | II-1 | II | LC387075 |
| MAFF 301885 | ++ | + | II | II-1 | II | LC387076 |
| MAFF 301886 | ++ | + | II | II-1 | II | LC387077 |
| MAFF 301887 | ++ | + | II | II-1 | II | LC387078 |
| MAFF 301888 | ++ | + | II | II-1 | II | LC387079 |
| MAFF 301889 | ++ | + | II | II-1 | II | LC387080 |
| MAFF 301890 | + | ++ | I | I | I | LC387081 |
| MAFF 301891 | ++ | + | II | II-1 | II | LC387082 |
| MAFF 301893 | + | ++ | I | I | I | LC387083 |
| MAFF 301894 | + | ++ | I | I | I | LC387084 |
| MAFF 301895 | + | ++ | I | I | I | LC387085 |
| MAFF 301896 | + | ++ | I | I | I | LC387086 |
| MAFF 301897 | ++ | + | II | II-1 | II | LC387087 |
| MAFF 301898 | ++ | + | II | II-1 | II | LC387088 |
| MAFF 301899 | + | ++ | I | I | I | LC387089 |
| MAFF 301900 | ++ | + | II | II-1 | II | LC387090 |
| MAFF 301901 | + | ++ | I | I | I | LC387091 |
| MAFF 301902 | + | ++ | I | I | I | LC387092 |
| MAFF 301903 | + | ++ | I | I | I | LC387093 |
| MAFF 301904 | ++ | + | II | II-1 | II | LC387094 |
| MAFF 301905 | ++ | + | II | II-1 | II | LC387095 |
| MAFF 301906 | ++ | + | II | II-1 | II | LC387096 |
| MAFF 301907 | ++ | + | II | II-1 | II | LC387097 |
| MAFF 301908 | ++ | + | II | II-1 | II | LC387098 |
| MAFF 301909 | ++ | + | II | II-1 | II | LC387099 |
| MAFF 301910 | ++ | + | II | II-1 | II | LC387100 |
| MAFF 301911 | ++ | + | II | II-1 | II | LC387101 |
| MAFF 301912 | ++ | + | II | II-1 | II | LC387102 |
| MAFF 301913 | ++ | + | II | II-1 | II | LC387103 |
| MAFF 301914 | + | ++ | I | I | I | LC387104 |
| MAFF 301915 | ++ | + | II | II-1 | II | LC387105 |
| MAFF 301916 | ++ | + | II | II-1 | II | LC387106 |
| MAFF 301917 | + | ++ | I | I | I | LC387107 |
| MAFF 301918 | + | ++ | I | I | I | LC387108 |
| MAFF 301919 | + | ++ | I | I | I | LC387109 |
| MAFF 301920 | ++ | + | II | II-1 | II | LC387110 |
| MAFF 301921 | ++ | + | II | II-1 | II | LC387111 |
| MAFF 301922 | ++ | + | II | II-1 | II | LC387112 |
| MAFF 301923 | – | – | III | II-2 | II | LC387113 |
| MAFF 301924 | – | – | III | II-2 | II | LC387114 |
| MAFF 301925 | ++ | + | II | II-1 | II | LC387115 |
| MAFF 301926 | + | ++ | I | I | I | LC387116 |
| MAFF 301927 | + | ++ | I | I | I | LC387117 |
| MAFF 301928 | ++ | + | II | II-1 | II | LC387118 |
| MAFF 301929 | ++ | + | II | II-1 | II | LC387119 |
| MAFF 301930 | ++ | + | II | II-1 | II | LC387120 |

| | | | | | | |
|---|---|---|---|---|---|---|
| MAFF 301931 | + | ++ | I | I | I | LC387121 |
| MAFF 301933 | ++ | + | II | II-1 | II | LC387122 |
| MAFF 301934 | ++ | + | II | II-1 | II | LC387123 |
| MAFF 301935 | ++ | + | II | II-1 | II | LC387124 |
| MAFF 301936 | ++ | + | II | II-1 | II | LC387125 |
| MAFF 301937 | ++ | + | II | II-1 | II | LC387126 |
| MAFF 301938 | + | ++ | I | I | I | LC387127 |
| MAFF 301939 | ++ | + | II | II-1 | II | LC387128 |
| MAFF 301940 | ++ | + | II | II-1 | II | LC387129 |
| MAFF 301941 | + | ++ | I | I | I | LC387130 |
| MAFF 301942 | ++ | + | II | II-1 | II | LC387131 |
| MAFF 301943 | – | – | III | II-2 | II | LC387132 |
| MAFF 301944 | ++ | + | II | II-1 | II | LC387133 |
| MAFF 301945 | – | – | III | II-2 | II | LC387134 |
| MAFF 301946 | ++ | + | II | II-1 | II | LC387135 |
| MAFF 301949 | + | ++ | I | I | I | LC387136 |
| MAFF 301950 | ++ | + | II | II-1 | II | LC387137 |
| MAFF 301952 | ++ | + | II | II-1 | II | LC387138 |
| MAFF 301954 | + | ++ | I | I | I | LC387139 |
| MAFF 302107 | ++ | + | II | II-1 | II | LC387140 |
| MAFF 302108 | + | ++ | I | I | I | LC387141 |
| MAFF 302109 | ++ | + | II | II-1 | II | LC387142 |
| MAFF 302110 | + | ++ | I | I | I | LC387143 |
| MAFF 302111 | + | ++ | I | I | I | LC387144 |
| MAFF 302112 | + | ++ | I | I | I | LC387145 |
| MAFF 302113 | ++ | + | II | II-1 | II | LC387146 |
| MAFF 302114 | ++ | + | II | II-1 | II | LC387147 |
| MAFF 302115 | ++ | + | II | II-1 | II | LC387148 |
| MAFF 302116 | ++ | + | II | II-1 | II | LC387149 |
| MAFF 302117 | ++ | + | II | II-1 | II | LC387150 |
| MAFF 302129 | + | ++ | I | I | I | LC387151 |
| MAFF 302653 | ++ | + | II | II-1 | II | LC387152 |
| MAFF 302749 | + | ++ | I | I | I | LC387153 |
| MAFF 302750 | ++ | + | II | II-1 | II | LC387154 |
| MAFF 302773 | ++ | + | II | II-1 | II | LC387155 |
| MAFF 302774 | ++ | + | II | II-1 | II | LC387156 |
| MAFF 302811 | + | ++ | I | I | I | LC387157 |
| MAFF 302812 | + | ++ | I | I | I | LC387158 |
| MAFF 302813 | ++ | + | II | II-1 | II | LC387159 |
| MAFF 302814 | ++ | + | II | II-1 | II | LC387160 |
| MAFF 302815 | ++ | + | II | II-1 | II | LC387161 |
| MAFF 302816 | ++ | + | II | II-1 | II | LC387162 |
| MAFF 302817 | ++ | + | II | II-1 | II | LC387163 |
| MAFF 302818 | ++ | + | II | II-1 | II | LC387164 |
| MAFF 302961 | ++ | + | II | II-1 | II | LC387165 |
| MAFF 302962 | ++ | + | II | II-1 | II | LC387166 |
| MAFF 302963 | ++ | + | II | II-1 | II | LC387167 |
| MAFF 302964 | ++ | + | II | II-1 | II | LC387168 |
| MAFF 302965 | ++ | + | II | II-1 | II | LC387169 |
| MAFF 311005 | ++ | + | II | II-1 | II | LC387170 |
| MAFF 311033 | – | – | III | II-2 | II | LC387171 |
| MAFF 311034 | – | – | III | II-2 | II | LC387172 |
| MAFF 311115 | ++ | + | II | II-1 | II | LC387173 |
| MAFF 311116 | ++ | + | II | II-1 | II | LC387174 |

| | | | | | | |
|---|---|---|---|---|---|---|
| MAFF 311224 | + | ++ | I | I | I | LC387175 |
| MAFF 311520 | + | ++ | I | I | I | LC387176 |
| MAFF 311521 | ++ | + | II | II-1 | II | LC387177 |
| MAFF 311522 | ++ | + | II | II-1 | II | LC387178 |
| MAFF 311523 | ++ | + | II | II-1 | II | LC387179 |
| MAFF 311524 | ++ | + | II | II-1 | II | LC387180 |
| MAFF 311525 | + | ++ | I | I | I | LC387181 |
| MAFF 311526 | ++ | + | II | II-1 | II | LC387182 |
| MAFF 311527 | + | ++ | I | I | I | LC387183 |
| MAFF 311528 | ++ | + | II | II-1 | II | LC387184 |
| MAFF 311529 | ++ | + | II | II-1 | II | LC387185 |
| MAFF 311530 | + | ++ | I | I | I | LC387186 |
| MAFF 311531 | + | ++ | I | I | I | LC387187 |
| MAFF 311532 | ++ | + | II | II-1 | II | LC387188 |
| MAFF 311533 | + | ++ | I | I | I | LC387189 |
| MAFF 311534 | ++ | + | II | II-1 | II | LC387190 |
| MAFF 311535 | ++ | + | II | II-1 | II | LC387191 |
| MAFF 311536 | ++ | + | II | II-1 | II | LC387192 |
| MAFF 311537 | ++ | + | II | II-1 | II | LC387193 |
| MAFF 311538 | ++ | + | II | II-1 | II | LC387194 |
| MAFF 311539 | ++ | + | II | II-1 | II | LC387195 |
| MAFF 311635 | ++ | + | II | II-1 | II | LC387196 |
| MAFF 331057 | ++ | + | II | II-1 | II | LC387197 |
| MAFF 331058 | ++ | + | II | II-1 | II | LC387198 |
| MAFF 331059 | ++ | + | II | II-1 | II | LC387199 |
| MAFF 331060 | ++ | + | II | II-1 | II | LC387200 |
| MAFF 730152 | + | ++ | I | I | I | LC387201 |
| MAFF 730153 | ++ | + | II | II-1 | II | LC387202 |
| MAFF 730154 | ++ | + | II | II-1 | II | LC387203 |
| MAFF 730209 | ++ | + | II | II-1 | II | LC387204 |
| MAFF 730211 | ++ | + | II | II-1 | II | LC387205 |
| MAFF 730212 | ++ | + | II | II-1 | II | LC387206 |
| MAFF 730213 | ++ | + | II | II-1 | II | LC387207 |
| MAFF 730214 | ++ | + | II | II-1 | II | LC387208 |
| MAFF 730215 | ++ | + | II | II-1 | II | LC387209 |
| MAFF 730216 | ++ | + | II | II-1 | II | LC387210 |
| MAFF 730217 | ++ | + | II | II-1 | II | LC387211 |
| MAFF 730218 | ++ | + | II | II-1 | II | LC387212 |
| MAFF 730219 | ++ | + | II | II-1 | II | LC387213 |
| MAFF 730220 | ++ | + | II | II-1 | II | LC387214 |
| MAFF 730221 | ++ | + | II | II-1 | II | LC387215 |
| MAFF 730222 | ++ | + | II | II-1 | II | LC387216 |
| MAFF 730224 | ++ | + | II | II-1 | II | LC387217 |
| MAFF 730225 | ++ | + | II | II-1 | II | LC387218 |
| MAFF 730243 | + | ++ | I | I | I | LC387219 |
| MAFF 730244 | + | ++ | I | I | I | LC387220 |
| MAFF 730245 | + | ++ | I | I | I | LC387221 |
| MAFF 730277 | ++ | + | II | II-1 | II | LC387222 |
| MAFF 730278 | ++ | + | II | II-1 | II | LC387223 |
| MAFF 730279 | ++ | + | II | II-1 | II | LC387224 |
| MAFF 730281 | ++ | + | II | II-1 | II | LC387225 |
| MAFF 810017 | ++ | + | II | II-1 | II | LC387226 |
| MAFF 810018 | ++ | + | II | II-1 | II | LC387227 |
| MAFF 810019 | ++ | + | II | II-1 | II | LC387228 |

| | | | | | | |
|---|---|---|---|---|---|---|
| MAFF 810020 | ++ | + | II | II-1 | II | LC387229 |
| MAFF 810021 | ++ | + | II | II-1 | II | LC387230 |
| MAFF 810022 | ++ | + | II | II-1 | II | LC387231 |
| MAFF 810023 | ++ | + | II | II-1 | II | LC387232 |
| MAFF 810024 | ++ | + | II | II-1 | II | LC387233 |
| MAFF 810025 | ++ | + | II | II-1 | II | LC387234 |
| MAFF 810026 | ++ | + | II | II-1 | II | LC387235 |
| MAFF 810027 | ++ | + | II | II-1 | II | LC387236 |
| MAFF 810028 | ++ | + | II | II-1 | II | LC387237 |
| MAFF 810029 | ++ | + | II | II-1 | II | LC387238 |
| MAFF 810030 | ++ | + | II | II-1 | II | LC387239 |
| MAFF 810031 | ++ | + | II | II-1 | II | LC387240 |
| MAFF 810032 | ++ | + | II | II-1 | II | LC387241 |
| MAFF 810033 | ++ | + | II | II-1 | II | LC387242 |
| MAFF 810034 | ++ | + | II | II-1 | II | LC387243 |
| MAFF 810035 | + | ++ | I | I | I | LC387244 |

[a] AHL-production type I, strongly induces violacein production in VIR07 (++) but weakly in CV026 (+). AHL-production type II, strongly induces violacein production in CV026 (++) but weakly in VIR07 (+). AHL-production type III, does not induce violacein production no induction in both CV026 and VIR07 (–).

[b] QS class I, produces 3-oxo-C8-HSL as a major AHL. QS class II-1, produces 3-oxo-C6-HSL as a major AHL. QS class II-2, produces a small amount of 3-oxo-C6-HSL.

[c] ExpI is divided into two clades (clade I and II) at an identity level of 75%. ExpI clade I is additionally divided into two subclades (clade I-1 and I-2) at an identity level of 95%.

Table S2. The production ratio of AHL extracted from the culture supernatant of *Pcc* strains

| Strain | Production ratio (%) | | | | | QS class |
|---|---|---|---|---|---|---|
| | 3-oxo-C6-HSL | C6-HSL | 3-oxo-C8-HSL | C8-HSL | 3-oxo-C10-HSL | |
| NBRC 3380 | 93 | 4 | 1 | 1 | 0 | II-1 |
| NBRC 3830 | 1 | 0 | 88 | 9 | 2 | I |
| NBRC 12380 | 91 | 8 | 1 | 0 | 0 | II-1 |
| MAFF 106567 | 1 | 2 | 74 | 20 | 2 | I |
| MAFF 106568 | 2 | 3 | 72 | 21 | 3 | I |
| MAFF 106569 | 1 | 3 | 74 | 20 | 2 | I |
| MAFF 106570 | 1 | 4 | 72 | 21 | 2 | I |
| MAFF 106571 | 2 | 2 | 72 | 22 | 3 | I |
| MAFF 106572 | 2 | 3 | 72 | 21 | 3 | I |
| MAFF 106573 | 1 | 1 | 76 | 19 | 2 | I |
| MAFF 106574 | 1 | 4 | 72 | 20 | 3 | I |
| MAFF 106575 | 96 | 3 | 1 | 0 | 0 | II-1 |
| MAFF 106576 | 1 | 1 | 75 | 21 | 2 | I |
| MAFF 106664 | 95 | 4 | 1 | 0 | 0 | II-1 |
| MAFF 106665 | 95 | 3 | 1 | 0 | 0 | II-1 |
| MAFF 211376 | 100 | 0 | 0 | 0 | 0 | II-2 |
| MAFF 211377 | 100 | 0 | 0 | 0 | 0 | II-2 |
| MAFF 211378 | 100 | 0 | 0 | 0 | 0 | II-2 |
| MAFF 211379 | 1 | 0 | 74 | 21 | 3 | I |
| MAFF 211380 | 1 | 1 | 77 | 18 | 3 | I |
| MAFF 211381 | 94 | 5 | 1 | 0 | 0 | II-1 |
| MAFF 211382 | 1 | 0 | 79 | 16 | 3 | I |
| MAFF 211383 | 1 | 0 | 80 | 16 | 3 | I |
| MAFF 211384 | 1 | 1 | 79 | 16 | 3 | I |
| MAFF 211385 | 1 | 2 | 77 | 17 | 3 | I |
| MAFF 211386 | 100 | 0 | 0 | 0 | 0 | II-2 |
| MAFF 211703 | 98 | 0 | 1 | 0 | 0 | II-1 |
| MAFF 212078 | 96 | 3 | 1 | 0 | 0 | II-1 |
| MAFF 212079 | 94 | 5 | 1 | 0 | 0 | II-1 |
| MAFF 212080 | 96 | 2 | 1 | 1 | 0 | II-1 |
| MAFF 212081 | 96 | 2 | 1 | 0 | 0 | II-1 |
| MAFF 212082 | 95 | 4 | 1 | 0 | 0 | II-1 |
| MAFF 212083 | 97 | 1 | 1 | 0 | 0 | II-1 |
| MAFF 301048 | 1 | 1 | 81 | 15 | 2 | I |
| MAFF 301049 | 95 | 3 | 1 | 1 | 0 | II-1 |
| MAFF 301050 | 95 | 4 | 1 | 0 | 0 | II-1 |
| MAFF 301051 | 93 | 6 | 1 | 0 | 0 | II-1 |
| MAFF 301052 | 2 | 1 | 79 | 16 | 3 | I |
| MAFF 301053 | 95 | 4 | 2 | 0 | 0 | II-1 |
| MAFF 301054 | 1 | 2 | 83 | 13 | 1 | I |
| MAFF 301056 | 97 | 2 | 1 | 0 | 0 | II-1 |
| MAFF 301281 | 96 | 3 | 1 | 0 | 0 | II-1 |
| MAFF 301282 | 96 | 3 | 1 | 0 | 0 | II-1 |
| MAFF 301283 | 1 | 3 | 74 | 19 | 3 | I |
| MAFF 301296 | 1 | 0 | 80 | 17 | 2 | I |
| MAFF 301297 | 94 | 4 | 2 | 0 | 0 | II-1 |
| MAFF 301298 | 88 | 11 | 1 | 0 | 0 | II-1 |
| MAFF 301299 | 94 | 4 | 1 | 0 | 0 | II-1 |
| MAFF 301300 | 94 | 4 | 1 | 1 | 0 | II-1 |
| MAFF 301301 | 95 | 4 | 1 | 0 | 0 | II-1 |

| | | | | | | |
|---|---|---|---|---|---|---|
| MAFF 301362 | 95 | 3 | 1 | 0 | 0 | II-1 |
| MAFF 301363 | 93 | 5 | 1 | 0 | 0 | II-1 |
| MAFF 301364 | 94 | 5 | 1 | 0 | 0 | II-1 |
| MAFF 301365 | 90 | 10 | 1 | 0 | 0 | II-1 |
| MAFF 301366 | 97 | 2 | 1 | 0 | 0 | II-1 |
| MAFF 301391 | 95 | 4 | 1 | 0 | 0 | II-1 |
| MAFF 301392 | 98 | 2 | 1 | 0 | 0 | II-1 |
| MAFF 301393 | 94 | 4 | 1 | 1 | 0 | II-1 |
| MAFF 301394 | 2 | 1 | 79 | 16 | 3 | I |
| MAFF 301395 | 95 | 3 | 1 | 0 | 0 | II-1 |
| MAFF 301396 | 1 | 2 | 81 | 14 | 2 | I |
| MAFF 301397 | 89 | 10 | 1 | 0 | 0 | II-1 |
| MAFF 301398 | 90 | 9 | 1 | 0 | 0 | II-1 |
| MAFF 301399 | 1 | 0 | 83 | 14 | 2 | I |
| MAFF 301400 | 1 | 2 | 75 | 19 | 2 | I |
| MAFF 301401 | 94 | 4 | 1 | 0 | 0 | II-1 |
| MAFF 301402 | 96 | 3 | 1 | 0 | 0 | II-1 |
| MAFF 301403 | 94 | 4 | 1 | 0 | 0 | II-1 |
| MAFF 301404 | 97 | 2 | 1 | 0 | 0 | II-1 |
| MAFF 301405 | 97 | 2 | 1 | 0 | 0 | II-1 |
| MAFF 301475 | 88 | 10 | 2 | 0 | 0 | II-1 |
| MAFF 301476 | 85 | 13 | 1 | 0 | 0 | II-1 |
| MAFF 301477 | 88 | 11 | 1 | 0 | 0 | II-1 |
| MAFF 301478 | 88 | 11 | 1 | 0 | 0 | II-1 |
| MAFF 301479 | 89 | 10 | 1 | 0 | 0 | II-1 |
| MAFF 301480 | 88 | 11 | 1 | 0 | 0 | II-1 |
| MAFF 301481 | 86 | 13 | 1 | 0 | 0 | II-1 |
| MAFF 301482 | 84 | 15 | 1 | 0 | 0 | II-1 |
| MAFF 301483 | 83 | 16 | 1 | 0 | 0 | II-1 |
| MAFF 301484 | 86 | 13 | 1 | 0 | 0 | II-1 |
| MAFF 301618 | 1 | 0 | 88 | 10 | 2 | I |
| MAFF 301619 | 1 | 0 | 80 | 18 | 1 | I |
| MAFF 301620 | 1 | 0 | 82 | 15 | 2 | I |
| MAFF 301645 | 84 | 15 | 1 | 0 | 0 | II-1 |
| MAFF 301646 | 82 | 16 | 1 | 0 | 0 | II-1 |
| MAFF 301647 | 84 | 14 | 1 | 0 | 0 | II-1 |
| MAFF 301648 | 82 | 17 | 1 | 0 | 0 | II-1 |
| MAFF 301649 | 86 | 13 | 1 | 0 | 0 | II-1 |
| MAFF 301650 | 88 | 11 | 1 | 0 | 0 | II-1 |
| MAFF 301651 | 88 | 11 | 1 | 0 | 0 | II-1 |
| MAFF 301652 | 88 | 12 | 1 | 0 | 0 | II-1 |
| MAFF 301653 | 86 | 13 | 1 | 0 | 0 | II-1 |
| MAFF 301654 | 87 | 12 | 1 | 0 | 0 | II-1 |
| MAFF 301655 | 85 | 13 | 1 | 0 | 0 | II-1 |
| MAFF 301656 | 80 | 19 | 1 | 0 | 0 | II-1 |
| MAFF 301865 | 79 | 19 | 1 | 0 | 0 | II-1 |
| MAFF 301867 | 80 | 20 | 0 | 0 | 0 | II-1 |
| MAFF 301868 | 79 | 19 | 1 | 0 | 0 | II-1 |
| MAFF 301869 | 85 | 13 | 2 | 0 | 0 | II-1 |
| MAFF 301871 | 86 | 13 | 1 | 0 | 0 | II-1 |
| MAFF 301872 | 90 | 10 | 0 | 0 | 0 | II-1 |
| MAFF 301873 | 92 | 7 | 1 | 0 | 0 | II-1 |
| MAFF 301874 | 86 | 14 | 0 | 0 | 0 | II-1 |
| MAFF 301875 | 1 | 0 | 83 | 15 | 1 | I |

| | | | | | | |
|---|---|---|---|---|---|---|
| MAFF 301876 | 90 | 9 | 1 | 0 | 0 | II-1 |
| MAFF 301877 | 93 | 7 | 1 | 0 | 0 | II-1 |
| MAFF 301878 | 90 | 9 | 1 | 0 | 0 | II-1 |
| MAFF 301879 | 100 | 0 | 0 | 0 | 0 | II-2 |
| MAFF 301880 | 86 | 13 | 1 | 0 | 0 | II-1 |
| MAFF 301881 | 100 | 0 | 0 | 0 | 0 | II-2 |
| MAFF 301882 | 89 | 11 | 1 | 0 | 0 | II-1 |
| MAFF 301883 | 1 | 0 | 87 | 11 | 1 | I |
| MAFF 301884 | 89 | 10 | 1 | 0 | 0 | II-1 |
| MAFF 301885 | 89 | 10 | 1 | 0 | 0 | II-1 |
| MAFF 301886 | 91 | 9 | 1 | 0 | 0 | II-1 |
| MAFF 301887 | 87 | 12 | 1 | 0 | 0 | II-1 |
| MAFF 301888 | 89 | 10 | 1 | 0 | 0 | II-1 |
| MAFF 301889 | 97 | 3 | 0 | 0 | 0 | II-1 |
| MAFF 301890 | 1 | 0 | 85 | 12 | 2 | I |
| MAFF 301891 | 93 | 6 | 1 | 0 | 0 | II-1 |
| MAFF 301893 | 1 | 0 | 82 | 15 | 2 | I |
| MAFF 301894 | 1 | 0 | 87 | 10 | 2 | I |
| MAFF 301895 | 1 | 0 | 86 | 11 | 1 | I |
| MAFF 301896 | 2 | 0 | 84 | 13 | 1 | I |
| MAFF 301897 | 96 | 4 | 0 | 0 | 0 | II-1 |
| MAFF 301898 | 94 | 5 | 1 | 0 | 0 | II-1 |
| MAFF 301899 | 1 | 0 | 84 | 14 | 2 | I |
| MAFF 301900 | 94 | 5 | 1 | 0 | 0 | II-1 |
| MAFF 301901 | 2 | 0 | 85 | 12 | 2 | I |
| MAFF 301902 | 1 | 0 | 86 | 11 | 1 | I |
| MAFF 301903 | 1 | 0 | 86 | 11 | 1 | I |
| MAFF 301904 | 92 | 7 | 1 | 0 | 0 | II-1 |
| MAFF 301905 | 94 | 6 | 1 | 0 | 0 | II-1 |
| MAFF 301906 | 97 | 3 | 0 | 0 | 0 | II-1 |
| MAFF 301907 | 96 | 4 | 0 | 0 | 0 | II-1 |
| MAFF 301908 | 97 | 3 | 0 | 0 | 0 | II-1 |
| MAFF 301909 | 94 | 6 | 1 | 0 | 0 | II-1 |
| MAFF 301910 | 94 | 6 | 0 | 0 | 0 | II-1 |
| MAFF 301911 | 94 | 5 | 1 | 0 | 0 | II-1 |
| MAFF 301912 | 95 | 5 | 0 | 0 | 0 | II-1 |
| MAFF 301913 | 93 | 6 | 1 | 0 | 0 | II-1 |
| MAFF 301914 | 1 | 0 | 86 | 12 | 1 | I |
| MAFF 301915 | 95 | 5 | 0 | 0 | 0 | II-1 |
| MAFF 301916 | 93 | 6 | 1 | 0 | 0 | II-1 |
| MAFF 301917 | 2 | 0 | 89 | 8 | 1 | I |
| MAFF 301918 | 2 | 0 | 81 | 15 | 2 | I |
| MAFF 301919 | 1 | 0 | 86 | 11 | 2 | I |
| MAFF 301920 | 96 | 4 | 1 | 0 | 0 | II-1 |
| MAFF 301921 | 93 | 6 | 1 | 0 | 0 | II-1 |
| MAFF 301922 | 94 | 5 | 1 | 0 | 0 | II-1 |
| MAFF 301923 | 100 | 0 | 0 | 0 | 0 | II-2 |
| MAFF 301924 | 100 | 0 | 0 | 0 | 0 | II-2 |
| MAFF 301925 | 91 | 8 | 1 | 0 | 0 | II-1 |
| MAFF 301926 | 2 | 0 | 87 | 10 | 2 | I |
| MAFF 301927 | 1 | 0 | 86 | 11 | 2 | I |
| MAFF 301928 | 94 | 5 | 1 | 0 | 0 | II-1 |
| MAFF 301929 | 94 | 5 | 1 | 0 | 0 | II-1 |
| MAFF 301930 | 93 | 7 | 1 | 0 | 0 | II-1 |

| | | | | | | |
|---|---|---|---|---|---|---|
| MAFF 301931 | 1 | 0 | 84 | 13 | 1 | I |
| MAFF 301933 | 94 | 5 | 1 | 0 | 0 | II-1 |
| MAFF 301934 | 95 | 4 | 1 | 0 | 0 | II-1 |
| MAFF 301935 | 95 | 4 | 1 | 0 | 0 | II-1 |
| MAFF 301936 | 94 | 5 | 1 | 0 | 0 | II-1 |
| MAFF 301937 | 94 | 5 | 1 | 0 | 0 | II-1 |
| MAFF 301938 | 1 | 0 | 84 | 13 | 2 | I |
| MAFF 301939 | 85 | 14 | 1 | 0 | 0 | II-1 |
| MAFF 301940 | 88 | 11 | 1 | 0 | 0 | II-1 |
| MAFF 301941 | 1 | 0 | 79 | 18 | 2 | I |
| MAFF 301942 | 91 | 9 | 1 | 0 | 0 | II-1 |
| MAFF 301943 | 100 | 0 | 0 | 0 | 0 | II-2 |
| MAFF 301944 | 91 | 8 | 1 | 0 | 0 | II-1 |
| MAFF 301945 | 100 | 0 | 0 | 0 | 0 | II-2 |
| MAFF 301946 | 89 | 11 | 1 | 0 | 0 | II-1 |
| MAFF 301949 | 1 | 0 | 75 | 22 | 2 | I |
| MAFF 301950 | 87 | 12 | 1 | 0 | 0 | II-1 |
| MAFF 301952 | 90 | 9 | 1 | 0 | 0 | II-1 |
| MAFF 301954 | 1 | 0 | 85 | 14 | 0 | I |
| MAFF 302107 | 80 | 17 | 3 | 0 | 0 | II-1 |
| MAFF 302108 | 2 | 0 | 73 | 23 | 2 | I |
| MAFF 302109 | 83 | 17 | 0 | 0 | 0 | II-1 |
| MAFF 302110 | 1 | 0 | 74 | 23 | 2 | I |
| MAFF 302111 | 1 | 0 | 78 | 19 | 2 | I |
| MAFF 302112 | 2 | 0 | 76 | 21 | 2 | I |
| MAFF 302113 | 85 | 15 | 0 | 0 | 0 | II-1 |
| MAFF 302114 | 84 | 16 | 0 | 0 | 0 | II-1 |
| MAFF 302115 | 83 | 17 | 0 | 0 | 0 | II-1 |
| MAFF 302116 | 81 | 19 | 0 | 0 | 0 | II-1 |
| MAFF 302117 | 90 | 9 | 1 | 0 | 0 | II-1 |
| MAFF 302129 | 2 | 0 | 73 | 23 | 2 | I |
| MAFF 302653 | 91 | 9 | 0 | 0 | 0 | II-1 |
| MAFF 302749 | 1 | 0 | 77 | 20 | 2 | I |
| MAFF 302750 | 86 | 13 | 1 | 0 | 0 | II-1 |
| MAFF 302773 | 88 | 11 | 1 | 0 | 0 | II-1 |
| MAFF 302774 | 89 | 10 | 1 | 0 | 0 | II-1 |
| MAFF 302811 | 1 | 0 | 81 | 16 | 2 | I |
| MAFF 302812 | 2 | 0 | 81 | 15 | 1 | I |
| MAFF 302813 | 88 | 11 | 1 | 0 | 0 | II-1 |
| MAFF 302814 | 91 | 8 | 1 | 0 | 0 | II-1 |
| MAFF 302815 | 90 | 9 | 1 | 0 | 0 | II-1 |
| MAFF 302816 | 89 | 10 | 1 | 0 | 0 | II-1 |
| MAFF 302817 | 89 | 11 | 0 | 0 | 0 | II-1 |
| MAFF 302818 | 88 | 11 | 1 | 0 | 0 | II-1 |
| MAFF 302961 | 85 | 15 | 0 | 0 | 0 | II-1 |
| MAFF 302962 | 87 | 13 | 0 | 0 | 0 | II-1 |
| MAFF 302963 | 83 | 16 | 1 | 0 | 0 | II-1 |
| MAFF 302964 | 85 | 15 | 0 | 0 | 0 | II-1 |
| MAFF 302965 | 91 | 8 | 1 | 0 | 0 | II-1 |
| MAFF 311005 | 89 | 10 | 1 | 0 | 0 | II-1 |
| MAFF 311033 | 100 | 0 | 0 | 0 | 0 | II-2 |
| MAFF 311034 | 100 | 0 | 0 | 0 | 0 | II-2 |
| MAFF 311115 | 89 | 10 | 1 | 0 | 0 | II-1 |
| MAFF 311116 | 85 | 14 | 1 | 0 | 0 | II-1 |

| | | | | | | |
|---|---|---|---|---|---|---|
| MAFF 311224 | 1 | 0 | 79 | 18 | 2 | I |
| MAFF 311520 | 1 | 0 | 77 | 20 | 2 | I |
| MAFF 311521 | 81 | 17 | 1 | 0 | 0 | II-1 |
| MAFF 311522 | 80 | 18 | 1 | 1 | 0 | II-1 |
| MAFF 311523 | 84 | 15 | 1 | 0 | 0 | II-1 |
| MAFF 311524 | 76 | 22 | 1 | 1 | 0 | II-1 |
| MAFF 311525 | 1 | 0 | 69 | 28 | 2 | I |
| MAFF 311526 | 86 | 13 | 1 | 0 | 0 | II-1 |
| MAFF 311527 | 1 | 0 | 77 | 20 | 2 | I |
| MAFF 311528 | 74 | 26 | 0 | 0 | 0 | II-1 |
| MAFF 311529 | 82 | 16 | 1 | 1 | 0 | II-1 |
| MAFF 311530 | 1 | 0 | 71 | 26 | 2 | I |
| MAFF 311531 | 1 | 0 | 72 | 25 | 2 | I |
| MAFF 311532 | 82 | 16 | 1 | 1 | 0 | II-1 |
| MAFF 311533 | 1 | 0 | 77 | 20 | 1 | I |
| MAFF 311534 | 92 | 7 | 1 | 0 | 0 | II-1 |
| MAFF 311535 | 76 | 22 | 1 | 1 | 0 | II-1 |
| MAFF 311536 | 79 | 19 | 1 | 1 | 0 | II-1 |
| MAFF 311537 | 75 | 23 | 1 | 1 | 0 | II-1 |
| MAFF 311538 | 78 | 19 | 2 | 1 | 0 | II-1 |
| MAFF 311539 | 86 | 12 | 1 | 0 | 0 | II-1 |
| MAFF 311635 | 86 | 13 | 1 | 0 | 0 | II-1 |
| MAFF 331057 | 80 | 18 | 1 | 1 | 0 | II-1 |
| MAFF 331058 | 78 | 20 | 1 | 1 | 0 | II-1 |
| MAFF 331059 | 88 | 11 | 1 | 0 | 0 | II-1 |
| MAFF 331060 | 80 | 19 | 1 | 0 | 0 | II-1 |
| MAFF 730152 | 1 | 0 | 76 | 21 | 2 | I |
| MAFF 730153 | 81 | 17 | 1 | 0 | 0 | II-1 |
| MAFF 730154 | 89 | 9 | 1 | 0 | 0 | II-1 |
| MAFF 730209 | 79 | 21 | 0 | 0 | 0 | II-1 |
| MAFF 730211 | 67 | 32 | 1 | 0 | 0 | II-1 |
| MAFF 730212 | 74 | 15 | 1 | 9 | 0 | II-1 |
| MAFF 730213 | 74 | 25 | 1 | 0 | 0 | II-1 |
| MAFF 730214 | 84 | 15 | 1 | 1 | 0 | II-1 |
| MAFF 730215 | 90 | 9 | 1 | 0 | 0 | II-1 |
| MAFF 730216 | 85 | 15 | 0 | 0 | 0 | II-1 |
| MAFF 730217 | 82 | 16 | 1 | 1 | 0 | II-1 |
| MAFF 730218 | 82 | 16 | 1 | 1 | 0 | II-1 |
| MAFF 730219 | 84 | 15 | 1 | 1 | 0 | II-1 |
| MAFF 730220 | 84 | 15 | 1 | 0 | 0 | II-1 |
| MAFF 730221 | 84 | 14 | 1 | 1 | 0 | II-1 |
| MAFF 730222 | 91 | 8 | 1 | 0 | 0 | II-1 |
| MAFF 730224 | 94 | 6 | 0 | 0 | 0 | II-1 |
| MAFF 730225 | 90 | 9 | 1 | 0 | 0 | II-1 |
| MAFF 730243 | 1 | 0 | 82 | 15 | 2 | I |
| MAFF 730244 | 1 | 0 | 84 | 12 | 2 | I |
| MAFF 730245 | 1 | 0 | 85 | 11 | 2 | I |
| MAFF 730277 | 92 | 7 | 1 | 0 | 0 | II-1 |
| MAFF 730278 | 91 | 8 | 1 | 0 | 0 | II-1 |
| MAFF 730279 | 92 | 7 | 1 | 0 | 0 | II-1 |
| MAFF 730281 | 88 | 11 | 1 | 0 | 0 | II-1 |
| MAFF 810017 | 91 | 8 | 1 | 0 | 0 | II-1 |
| MAFF 810018 | 89 | 9 | 1 | 0 | 0 | II-1 |
| MAFF 810019 | 91 | 8 | 1 | 0 | 0 | II-1 |

| | | | | | | |
|---|---|---|---|---|---|---|
| MAFF 810020 | 90 | 9 | 1 | 0 | 0 | II-1 |
| MAFF 810021 | 91 | 8 | 1 | 0 | 0 | II-1 |
| MAFF 810022 | 90 | 9 | 1 | 0 | 0 | II-1 |
| MAFF 810023 | 90 | 8 | 1 | 0 | 0 | II-1 |
| MAFF 810024 | 89 | 10 | 1 | 0 | 0 | II-1 |
| MAFF 810025 | 92 | 7 | 1 | 0 | 0 | II-1 |
| MAFF 810026 | 89 | 9 | 1 | 0 | 0 | II-1 |
| MAFF 810027 | 92 | 8 | 1 | 0 | 0 | II-1 |
| MAFF 810028 | 90 | 9 | 1 | 0 | 0 | II-1 |
| MAFF 810029 | 91 | 8 | 1 | 0 | 0 | II-1 |
| MAFF 810030 | 92 | 7 | 1 | 0 | 0 | II-1 |
| MAFF 810031 | 90 | 9 | 1 | 0 | 0 | II-1 |
| MAFF 810032 | 90 | 9 | 1 | 0 | 0 | II-1 |
| MAFF 810033 | 93 | 6 | 1 | 0 | 0 | II-1 |
| MAFF 810034 | 89 | 9 | 1 | 0 | 0 | II-1 |
| MAFF 810035 | 1 | 0 | 83 | 14 | 2 | I |
